# Strong Vaccine-Induced CD8 T-Cell Responses Have Cytolytic Function in a Chimpanzee Clearing HCV Infection

**DOI:** 10.1371/journal.pone.0095103

**Published:** 2014-04-16

**Authors:** Babs E. Verstrepen, Ernst J. Verschoor, Zahra C. Fagrouch, Petra Mooij, Natasja G. de Groot, Ronald E. Bontrop, Willy M. Bogers, Jonathan L. Heeney, Gerrit Koopman

**Affiliations:** 1 Department of Virology, Biomedical Primate Research Centre, Rijswijk, The Netherlands; 2 Department of Comparative Genetics and Refinement, Biomedical Primate Research Centre, Rijswijk, The Netherlands; 3 Department of Veterinary Medicine, University of Cambridge, Cambridge, United Kingdom; University of Montreal, Canada

## Abstract

A single correlate of effective vaccine protection against chronic HCV infection has yet to be defined. In this study, we analyzed T-cell responses in four chimpanzees, immunized with core-E1-E2-NS3 and subsequently infected with HCV1b. Viral clearance was observed in one animal, while the other three became chronically infected. In the animal that cleared infection, NS3-specific CD8 T-cell responses were observed to be more potent in terms of frequency and polyfunctionality of cytokine producing cells. Unique to this animal was the presence of killing-competent CD8 T-cells, specific for NS3_1258–1272_, being presented by the chimpanzee MHC class I molecule Patr-A*03∶01, and a high affinity recognition of this epitope. In the animals that became chronically infected, T-cells were able to produce cytokines against the same peptide but no cytolysis could be detected. In conclusion, in the animal that was able to clear HCV infection not only cytokine production was observed but also cytolytic potential against specific MHC class I/peptide-combinations.

## Introduction

Hepatitis C virus (HCV) infection is characterized by a high propensity for development of chronic infection, which typically manifests as asymptomatic for a long period of time. However, over decades the virus causes subtle, but cumulatively irreversible, hepatic damage. A vaccine that could prevent development of persistent HCV infection would therefore be of great clinical benefit.

The chimpanzee is the only validated animal model of HCV infection [1], [2]. The dilemma that biomedical research in non-human primates, and chimpanzees in particular, is inevitably associated with low animal numbers and limiting statistical analysis, has been discussed elsewhere [3]. Nevertheless, we believe that results from in depth immune-profiling of immunized chimpanzees may provide new insights into immune mechanisms operating in the early phase after infection and as such is important for optimal vaccine development in the future.

Spontaneous HCV clearance has been associated with the presence of broad and strong T-cell responses in both humans and chimpanzees [4]–[7]. Moreover, specific memory T-cell responses often correlate with early clearance after HCV reinfection [8]–[10]. These data imply that T-cell-based vaccines may facilitate HCV clearance, protect from viral persistence and thus from HCV-related disease progression.

For this reason, several HCV T-cell vaccine-candidates have been evaluated. Some of these vaccine-strategies induced strong T-cell responses however, no correlation was observed between either the magnitude or the breadth of vaccine-induced T-cell responses, and viral clearance. The lytic capacity of vaccine-induced CD8 T-cells, in the context of MHC class-I presentation was not previously evaluated in relation to viral clearance.

Previously, we reported partial control of an HCV 1b challenge after a DNA prime-MVA-boost vaccine strategy, targeting HCV core, E1, E2 and NS3 in chimpanzees [11]. In these animals, neither (neutralizing) antibody responses nor *ex vivo* cytokine production or proliferative responses were uniquely associated with control of HCV infection. Therefore, the current study aimed to identify potential mechanisms of protection via detailed functional characterization of the T-cell responses against the most dominantly recognized antigen, NS3 [11].

We report that the animal that cleared infection had a high percentage of polyfunctional cytokine producing CD4 and CD8 T-cells against NS3-peptides. Moreover, a strong cytolytic T-cell (CTL) response against epitope NS3_1258–1272_ was identified, which was uniquely presented in the context of the chimpanzee MHC class I molecule Patr-A*03∶01.

## Materials and Methods

### Ethics statement

This retrospective study was performed on cryopreserved PBMCs, isolated from animals that were part of an HCV vaccine-efficacy study [11]. As previously described, the preceding study had been performed in six purpose bred, naive mature chimpanzees (*Pan troglodytes*) that were housed at the Biomedical Primate Research Centre (BPRC), Rijswijk, The Netherlands, according to international guidelines for non-human primate care and use (The European Council Directive 86/609/EEC, and Convention ETS 123, including the revised Appendix A). The Institutional Animals Care and Use Committee (DEC-BPRC) approved study protocols (#426) according to strict international ethical and scientific standards and guidelines. The qualification of the members of this committee, including their independence from a research institute, is stated in the “Wet op de Dierproeven” (1996). The experiment can only be performed after a positive recommendation from this ethical committee. Permission for projects on non-human primates is requested in an animal ethical committee form. This form is discussed in the ethical committee and, if approved, a written permission is sent to the project leader and a copy to the director of the BPRC, who holds licence to perform animal experimentation. The project was monitored by a qualified independent veterinarian, specifically regarding the ethical issues of the projects.

The animals were in good physical health with normal biochemical and hematological values. The animals were socially housed in a BSL3-facility with spacious cages, and were provided with commercial food pellets supplemented with appropriate treats. Drinking water was provided *ad libitum.* Environmental enrichment was provided daily and suffering of the animals was alleviated wherever possible. In 2002, the European Council banned the use of apes for biomedical research in Europe. Yet, this was decided after the initiation of the original vaccine-study described by Rollier *et. al.* [11]. For this reason, the European Council approved continuation of this particular study. The following years, all chimpanzees that were housed at the BPRC were outplaced to public zoos or animal sanctuaries. Before 2001, the use of chimpanzees for biomedical research was highly restricted and regulated, it was not allowed to perform terminal experiments on these animals [12].

### Peptide-pools

For stimulation and expansion of HCV-specific cells, two different NS3-peptide-pools were compiled: NS3_vaccine_ was a mixture of 15-mer peptides with 11 amino acids overlap, covering the NS3-sequence of the vaccine constructs; NS3_challenge_ was a mixture of 15-mer peptides with 11 amino acids overlap covering the NS3-variants observed in the inoculum. Identification of NS3 epitopes within NS3 was first performed via the matrix setup [13] and confirmed in a second assay using individual peptides (peptide-sequences are listed in Table S1).

### In vitro expansion of NS3-specific T-cell lines

Cells isolated before infection were expanded and tested for response against peptide-pool NS3_vaccine_, whereas cells isolated after challenge were tested against peptide-pool NS3_challenge_. PBMCs isolated at the day of challenge were stimulated in two separate cultures with either NS3_vaccine_ or NS3_challenge_.

After thawing, PBMCs were washed and resuspended in culture medium, R20 (RPMI, Invitrogen, CA, USA) supplemented with 20% FCS (MP medicals, Solon, OH, USA), pen/strep (100 U/ml/100 µg/ml) (Invitrogen) and L-glutamine (2 mM) (Invitrogen)) and cultured at a final density of 10 million cells per ml in a 24 well culture plate (Greiner bio-one). The cultures were initiated in R20 containing 5 ng/ml IL-7 (Peprotech) and NS3-peptide-pool (5 µg/ml per peptide). After overnight incubation, 1 ml R20 containing IL-7 (10 ng/ml) and IL-2 (Proleukin Chiron, final concentration 10 IU/ml) was added per well. Cultures were checked daily for general condition and depending on cell density, fresh R20 IL-2/IL-7 was added or cultures were split. After 12 days, the expanded cells were collected and resuspended in fresh R20 for further analysis.

### T-cell epitope mapping

The cytokine production profile of HCV-specific T-cell lines was determined by intracellular cytokine staining (ICS) after restimulation with NS3-peptides. For T-cell epitope mapping 0.2×10^6^ expanded cells were either restimulated with NS3-peptide-pool (1 µg/ml/peptide) or without NS3-peptide, in medium containing co-stimulatory αCD28 and αCD49d molecules (2 µg each, BD-Biosciences) and 5% FCS (MP-Biomedicals). After 2 hrs, Brefeldin A (Golgiplug 1∶1000, BD-Biosciences) was added and 16 hours later the surface markers were stained with a panel of fluorochrome labeled antibodies, containing CD3-PacificBlue (clone SP-34-2, (BD-Pharmingen), CD14-PE-TexasRed (clone RMO52, BeckmanCoulter), CD20-PE-TexasRed (clone B9E9, BeckmanCoulter), CD4-PE-Cy7 (clone SK3, BD-Pharmingen) and CD8-APC-H7 (clone SK1, BD-Pharmingen). Stained cells were fixed and permeabilized (Cytofix/Cytoperm, BD Biosciences), followed by staining of accumulated intracellular cytokines with IFNγ-APC (clone B27, BD Pharmingen), IL-2-PE (clone MQ1-17H12, BD Pharmingen) and TNFα-FITC (clone MAb11, BD Pharmingen). Cell staining was analyzed on a FACSAria (BD Bioscience) and DIVA software Version 6.1.1.

To evaluate antigen sensitivity, T-cell lines were restimulated with increasing concentrations of individual peptide ranging from 0.05 to 10 µg/ml before intracellular cytokine production was measured.

### MHC-class-I genotyping

Animals were genotyped for their MHC-class-I repertoire as previously described [14] (Table 1).

**Table 1:** MHC class I genotypes of the chimpanzees.

	Patr class I alleles			
	Patr-A		Patr-B	
Vac1	A*03∶01	A*04∶04	B*02∶01	B*03∶01
Vac2	A*06∶01	A*04∶01	B*01∶01	B*03∶01
Vac3	A*09∶01	-A	B*01∶01	B*02∶01
Vac4	A*09∶01	A*0101	B*01∶01	B*16∶0101

A Vac3 is likely to be homozygous A*0901.

### Non-radioactive cytotoxicity assay

NS3-peptide-specific T-cell lines were tested for their capacity to kill peptide-loaded target cells, using a new non-radioactive cytotoxicity assay. In this assay, a panel of matched *Patr* MHC-class-I expressing transfectants, either constructed in 721.221 cells (kindly provided by C. Walker, Center for Vaccines and Immunity, The Ohio State University) or K562 [15] were used as target cells. In brief, 3×10^6^ MHC-matched transfected target cells were labeled with 1 µM CFSE (CFSE^low^) (Fluka) and another 3×10^6^ MHC-matched transfected target cells, expressing the same class-I molecule were labeled with 8 µM CFSE (CFSE^high^). Subsequently, CFSE^low^ cells were incubated with medium alone, whereas, CFSE^high^ target cells were pulsed with HCV-peptide (8 µg/ml) for 2 hrs at 37°C. Excess peptide was removed by washing before mixing CFSE^high^ and CFSE^low^ with 1×10^6^ NS3-specific expanded T-cells (ET ratio 10∶1). The mixed target and effector cells were cocultured for 16 hrs, after which CFSE staining was analyzed using a FACSAria (BD Bioscience) and DIVA software Version 6.1.1.

Specific lysis was calculated using the following formula;
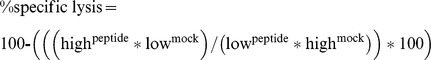



and lysis was considered positive when exceeding 25%.

### Peptide-binding assay

A cell-based peptide-binding-competition-assay was performed as previously described [15]. In this assay, peptides are tested for binding to transfected cell lines expressing chimpanzee class-I molecules, while competing with a biotin-labeled reference-peptide. In a 96 wells plate, 100.000 MHC class-I transfected K562 or 721.221 cells were incubated with 500nM of the biotinylated-indicator-peptide (in case of A*03∶01 ATALECVYK was used, and in case of B*01∶01 LSDMHLCSI, in which C indicates the position of the biotin-labeled cysteine) and 0.05 to 100 nM of the peptide of interest, for instance p59 (NS3_1258–1272_), LGFGAYMSK or AATLGFGAY. Peptide KGGLRPRAG, predicted to have no or a very low binding affinity to MHC class I molecules based on the incorporation of glycines on the anchor positions, was used as control. After overnight incubation, excess peptide was removed by washing and indicator-peptide bound to cells was quantified using the DELFIA-system and the Victor3 1420 multilabel counter (Perkin Elmer). The binding affinity of the indicator peptide was verified using its unlabeled peptide as competitor, this is referred to as standardized competitor [15].

## Results

### Characterization of NS3 specific CD4 and CD8 T-cell cytokine responses

We previously reported [11] an HCV-vaccine evaluation-study in chimpanzees, in which 4 out of 4 immunized animals became viremic shortly after challenge with HCV 1b J4. However, at a later stage, animal Vac1 cleared HCV infection while the three other animals, Vac2, Vac3 and Vac4 became chronically infected. As reported previously, IFNγ release, *ex vivo* lymphoproliferative responses, Th1/Th2 cytokine release patterns, and HCV-specific antibodies were comparable amongst the four animals [11]. NS3 was the immunodominant antigen in all four animals, while the responses against core, E1 and E2 were not only less intense, but also largely absent in Vac1, the animal which was protected against chronic infection. Therefore, functional analysis of the cellular responses was focused on the immunodominant NS3 antigen [11].

**Figure 1:**
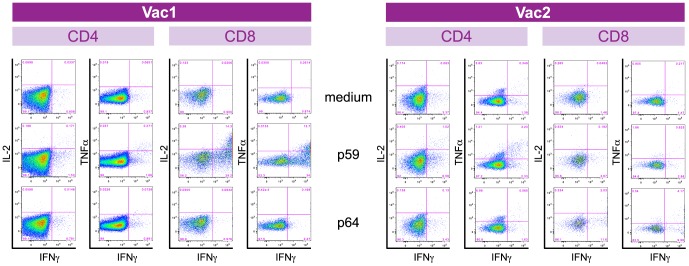
Evaluation of peptide-specific IL-2, IFNγ and TNFα expression in CD4 and CD8 T-cells. A representative example of the analysis performed in Vac1 (left panel) and Vac2 (right panel), two weeks after the final vaccine boost, is shown. PBMC were first expanded for a 12 day period using a pool of all NS3 peptides, restimulated with individual peptides (medium alone, p59 or p64) and analyzed for induction of IFNγ, IL-2 and TNFα cytokine expression by expanded CD4 and expanded CD8 cells, using the gating strategy described in Figure S1. The numbers in the quadrants, represent the percentage of positive cells calculated from the parent-population.

After 12 days of culturing in the presence of a pool of NS3 peptides, T-cell lines were restimulated with individual peptides and analyzed for intracellular expression of IFNγ, IL-2 and TNFα using the gating strategy shown in Figure S1. Cells within the lymphocyte gate that were CD3 positive, and CD14 and CD20 negative were selected and subsequently IL-2, IFNγ and TNFα single, dual or triple cytokine expression was analyzed in the CD4 and CD8 T-cell subsets within the T-cell lines. Cytokine expression profiles varied per peptide as illustrated in Figure 1. For instance, only moderate cytokine induction was observed after peptide specific restimulation of expanded CD4 cells in Vac1. In contract, strong dual IFNγ and IL-2 expression was seen in expanded CD8 T-cells restimulated with peptide 59 (NS3_1258–1272_), while medium alone or peptide 64 (IRTGVRTITTGGPIT) did not induce cytokine production. In comparison, in Vac2 p59 only gave a marginal cytokine induction in expanded CD8 T-cells, while p64 induced moderate dual IFNγ/IL-2 and IFNγ/TNFa cytokine production (Figure 1). Instead, expanded CD4 T-cells in Vac2, showed stronger response towards p59.

**Figure 2:**
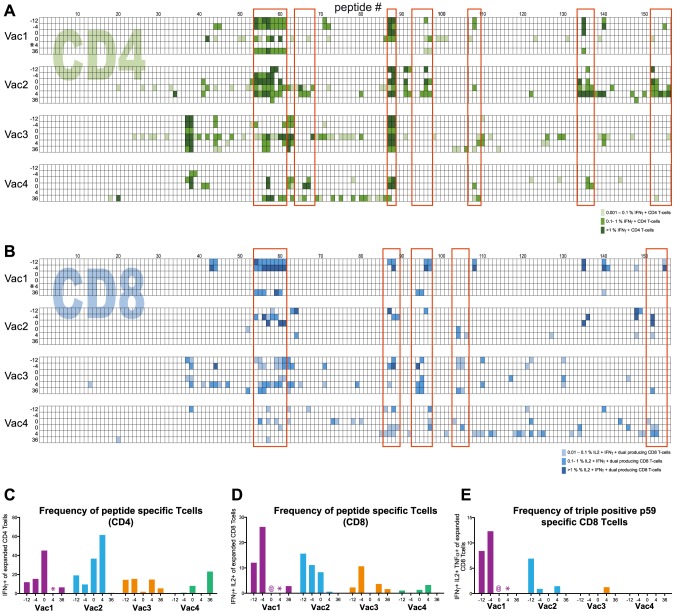
Heat map of NS3-peptide-specific T-cell responses in four immunized chimpanzees at different study time points. (A) Percentage of expanded CD4 T-cells producing IFNγ upon restimulation with individual peptides, subdivided into three categories; i.e. low response (light green; 0.001%–0.1% specific IFNγ production), intermediate (green; 0.1–1% specific response), high (dark green;>1% specific response). (B) Percentage of expanded CD8 T-cells showing IL-2/IFNγ double cytokine production upon restimulation with individual peptide, subdivided into three categories; i.e. low response (light blue; 0.01%–0.1% specific IFNγproduction), intermediate (blue; 0.1–1% specific response), high (dark blue; > 1% specific response). White areas indicate where no responses were detected. The red boxes highlight areas within NS3 that are broadly recognized. Per animal 6 time points of analysis are shown lined up beneath each other; -12) Two weeks following the 1st MVA boost, cells stimulated with NS3_vaccine_, -4) Four weeks following 2^nd^ MVA boost, cells stimulated with NS3_vaccine_, 0) Day of HCV infection, stimulation with NS3_vaccine_, 0) Day of HCV infection, stimulation with NS3_challenge_, and 4 and 36 weeks after challenge, stimulation with NS3_challenge_. The individual peptides tested are indicated at the top of the map by the numbers 1 to 156. The red boxes represent broadly recognized regions within NS3. Total frequency (all peptide responses combined) of (C) IFNγ production by expanded CD4 T-cells and (D) IFNγ/IL2 dual cytokine production by expanded CD8 T-cells per animal. (E) Frequency of p59 specific triple positive IL2/TNFα/IFNγ producing CD8 T-cells. The numbers on the X-axis represent the study week, relative to time of challenge. * Despite several attempts, frozen cells from Vac1 from 4 weeks following HCV infection did not respond to either NS3_vaccine_ or NS3_challenge_ peptide stimulation and no expansion could be achieved. @ Due to a high IFNγ background, peptide specific CD8 responses could not be detected

In this manner the responses against each individual NS3 peptide was investigated and the results are summarized in Figure 2 in the form of heat maps; showing the percentages of IFNγ producing CD4 (Figure 2(A)) and IFNγ/IL-2 dual cytokine producing CD8 (Figure 2(B)) T-cells in all four animals at all time points tested. In general, analysis of peptide responses by dual cytokine expression gives a clear-cut distinction between positive and negative responses, because of low background responses. However, for CD4 T-cells the number of dual cytokine expressing cells was often rather low, making it necessary to consider the total number of IFNγ cytokine producing cells. The peptide recognition profiles shown in Figures 2(A and B) indicate that some regions within the NS3 peptide sequence, indicated by red boxes, are broadly recognized amongst the animals. Other peptide specific responses were more restricted and only gave positive responses at some time points in one to two animals. No unique peptide response-pattern could be discerned for Vac1 that could explain vaccine induced viral clearance as compared to Vac2, 3, and 4. Moreover, no relation was observed between the number of peptides recognized and the course of infection (Table 2).

**Table 2:** Number of NS3-peptides that induce IFNγ production by CD4 T-cells or IL-2/IFNγ dual cytokine production by CD8 T-cells.

		TOTAL	PRE	POST
CD4	**Vac1**	**34**	**34**	**11**
	**Vac2**	**55**	**42**	**36**
	**Vac3**	**58**	**51**	**22**
	**Vac4**	**38**	**19**	**25**
CD8	**Vac1**	**22**	**20**	**8**
	**Vac2**	**22**	**20**	**6**
	**Vac3**	**35**	**22**	**27**
	**Vac4**	**44**	**27**	**25**

PRE, pre challenge. POST, post challenge.

Next, the sum of the % of cytokine producing cells induced by all 156 individual peptides was calculated for expanded CD4 and CD8 lines. This total frequency of peptide-specific IFNγ-producing expanded CD4 T-cells was comparable between Vac1 and Vac2, but somewhat lower in Vac3 and Vac4 (Figure 2(C)). After the last immunization (week -4), total peptide specific CD8 T-cell responses were higher in Vac1 as compared to the other animals (Figure 2(D)). Triple cytokine production, i.e. simultaneous expression of IFNγ, IL-2 and TNFα by expanded CD8 cells, was also higher in Vac1 and for instance observed in 12.3% of the p59-specific expanded CD8 T-cells 4 weeks prior to HCV infection, whereas this was less than 7% in Vac2, Vac3 and Vac4 (Figure. 2(E)).

In conclusion, we observed increased number of NS3 specific cytokine producing expanded CD8 T-cells after the last immunization, and especially more triple IFNγ, IL-2 and TNFα cytokine producing cells in the animal that cleared the virus, relative to those animals that became chronically infected.

### Cytolytic capacity of NS3 p59 specific CD8 T-cells in Vac1

Next, NS3-peptide-specific cells were evaluated for their capacity to kill peptide-loaded target cells. Similar to standard ^51^chromium release assays, T-cells were expanded prior to analysis [6], [16]. The expanded T-cells were tested for their lytic capacity for all individual peptides that were found to induce IL-2/IFNγ dual expression in expanded CD8 cells (Figure 2(B)) and was tested against a panel of MHC matched target cells [15] (Table 1).

**Figure 3:**
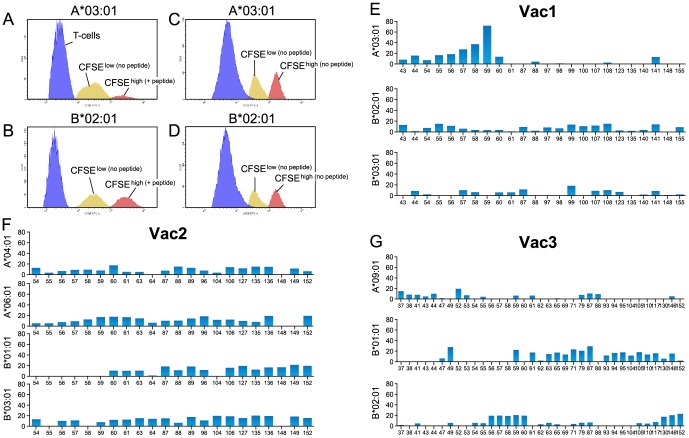
Cytolytic killing of CFSE-loaded target cells. (A) Specific lysis of NS3_1258–1272_-pulsed Patr-A*03∶01 target cells (red peak) in comparison with unpulsed Patr-A*03∶01 target cells (yellow peak) by NS3-peptide-pool expanded PBMC from Vac1 isolated 10 weeks after HCV 1bJ4 challenge (blue peak). (B) No specific lysis of NS3_1258–1272_-pulsed Patr-B*02∶01 target cells. (C) and (D) Control experiments showing no killing of unpulsed Patr-A*03∶01 or Patr-B*02∶01 target cells. (E,F,G) Percentage of lysis for all MHC class-I-peptide-combinations tested in Vac1, Vac2, Vac3 respectively.

Figure 3(A) illustrates specific killing of p59-pulsed CFSE^high^ relative to unpulsed CFSE^low^ Patr-A*03∶01 target cells, using expanded T-cells from Vac1, isolated 10 weeks after HCV challenge. As a control, no difference in ratio of the area under the curve was observed if both the CFSE^high^ and CFSE^ low^ Patr-A*03∶01 targets were unpulsed (Figure 3(C)), precluding non-specific toxicity due to labeling with higher CFSE concentration. Killing of target cells was only observed for particular peptide-MHC class I combinations. As shown in Figures 3(B and D), neither unpulsed nor p59-pulsed Patr-B*02∶01 targets were lysed by expanded cells from Vac1.

Amongst all peptides tested, i.e. 24, 22 and 35 in Vac1, Vac2 and Vac3 respectively, highly efficient killing was only observed against p59 in Vac1 and only when this peptide was presented by Patr-A*03∶01 (Figure 3(E to G)). Only the peptides partly overlapping with p59 gave some lysis, while none of the other MHC class-I-peptide-combinations exceeded 25% of specific killing. Due to the limited number of cells after expansion, Vac4 could not be tested.

Collectively, the data show that although stimulation with p59 can induce some level of cytokine production in all vaccinees, a p59-mediated cytolytic capacity was only observed in Vac1.

### NS3p59(_aa1258–1272)_ binds with high affinity to Patr-A*03∶01

**Figure 4:**
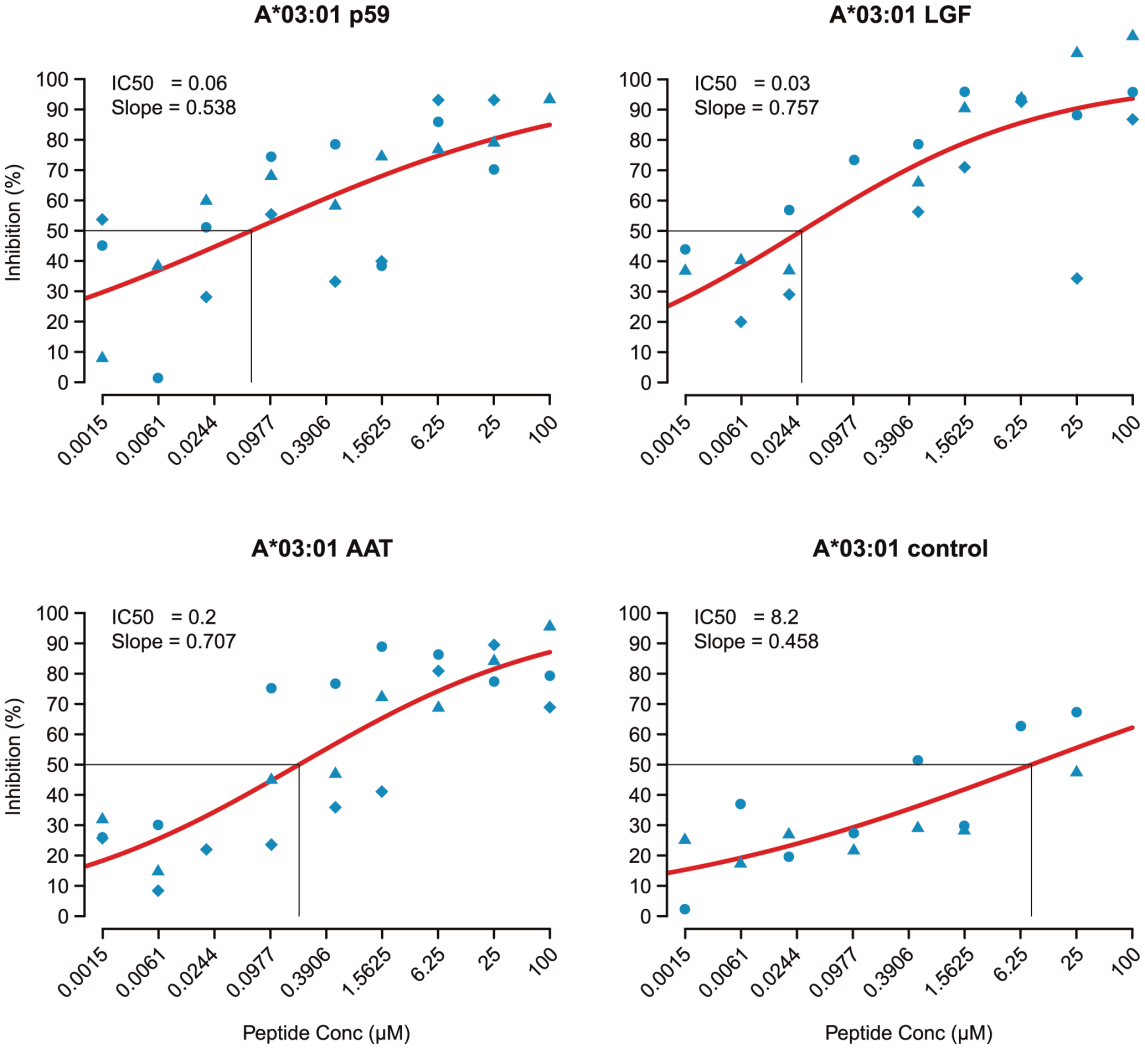
Dose-inhibition curves for selected NS3-peptides to Patr-A*03∶01. Binding affinity of selected NS3-peptides to Patr-A*03∶01, established by peptide binding competition assay. Patr-A*0301 cells were incubated with increasing concentrations of NS3 p59 (top left), LGFGAYMSK (LGF, top right), AATLGFGAY (AAT, bottom left), or KGGLRPRAG (control, bottom right) in the presence of biotin-labeled indicator peptide. Binding of indicator peptide was quantified by incubation with europium labeled streptavidin. Indicated is reduction in percentage of europium positive cells relative to cells incubated with indicator-peptide only. The indicated IC_50_ values (µM) are derived from the regression curves of three independent experiments in the case of p59, LGF and AAT, and of two individual experiments in the case of KGG. The IC_50_ values were estimated using non-linear least-squares regression with the “R” platform for statistical computing.

Peptide binding to MHC-class I is determined by the anchor-residues fitting into the B- and the F-pocket of the relevant allotype for most MHC class-I-molecules. Usually MHC class I molecules bind nonamer peptides, in which the p2-position of the peptide anchors into the B-pocket and the p9-position of the peptide anchors into the F-pocket. Peptide 59, which was observed to initiate cytolysis when presented in the context of Patr-A*03∶01, was scanned using the NetMHCpan version 2.8 prediction algorithm [17] against the MHC class I molecules present in the studied cohort (Table 1). It is known that peptides that bind, or are predicted to bind to MHC class I molecules with an affinity <500 nm are potential T-cell epitopes [18]–[20]. In p59 four potential nonamer peptides are present, AATLGFGAY, ATLGFGAYM, TLGFGAYMS, and LGFGAYMSK. The only combination that was predicted to bind with an affinity <500 nm was LGFGAYMSK in the context of Patr-A*03∶01. A cell-based peptide binding competition assay was performed to test the binding-affinity of p59, and the nonamer peptides AATLGFGAY and LGFGAYMSK to Patr-A*03∶01 and as control to Patr-B*01∶01. The peptide binding motif of Patr-A*03∶01 is defined as X(STA)XXXXXX(RK) and for Patr-B*01∶01 as (X(ST)XXXXXX(IL) [15], [21]. Figure 4 shows that all three NS3 peptides have a high binding affinity to Patr-A*03∶01, with IC_50_ values between 0.03 to 0.2 µM, which is higher compared to the control peptide (IC_50_ = 8.2 µM) and the standardized competitor peptide of Patr-A*03∶01 with IC_50_ = 1.5 µM [15]. Peptide LGFGAYMSK was found to have the highest binding affinity to Patr-A*03∶01. In contrast, only low binding affinity was observed for p59 in the context of Patr-B*01∶01 as compared to its standardized competitor (IC_50_ = 1.0 µM), whereas for the two nonamer peptides, no amenable regression curves could be drawn suggesting low or no binding affinity (not shown).

### T-cells of Vac1 respond at lower peptide concentration

**Figure 5:**
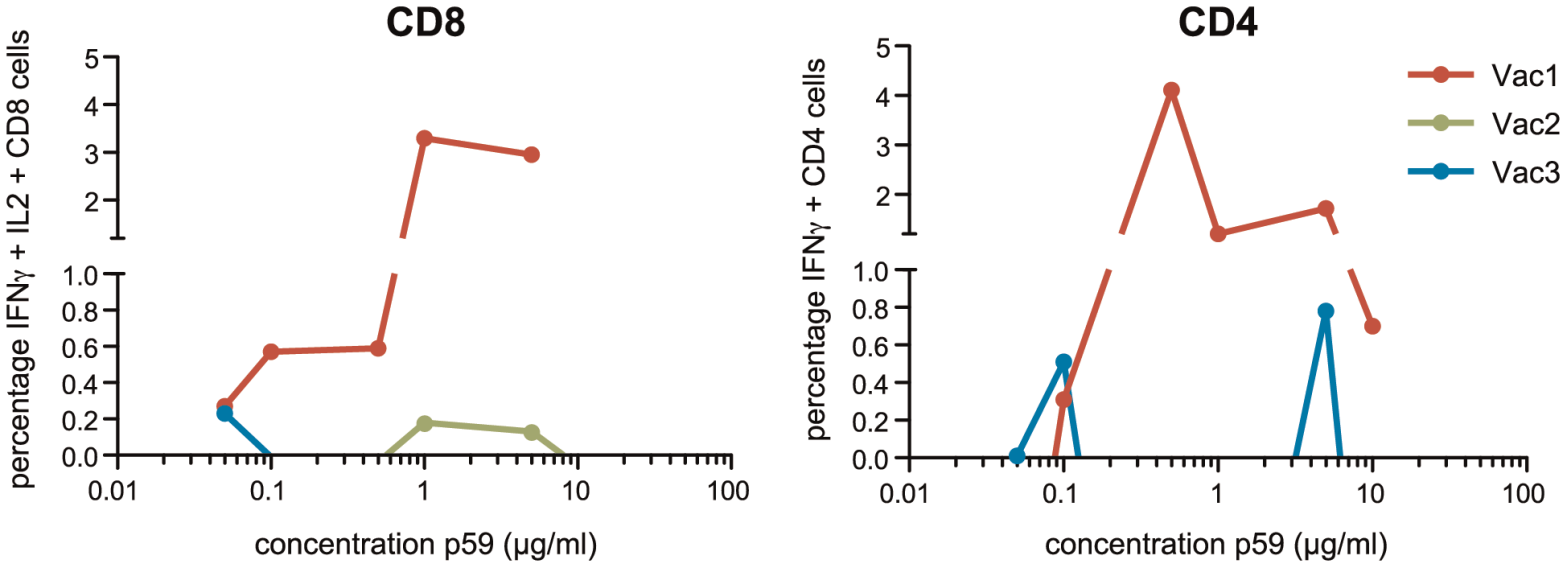
Antigen sensitivity of p59 specific cytokine induction. NS3-expanded T-cells from Vac1, Vac2 and Vac3, from 4 weeks prior to HCV challenge, were restimulated with a concentration range of p59 and evaluated for induction of IL-2/IFNγ expressing CD8 T-cells (left graph) and IFNγ expressing CD4 T-cells (right graph).

Antigen specific responses not only depend on the presentation of peptides by MHC class I molecules but also on the binding of the peptide/MHC complex to the T-cell-receptor (TCR). Therefore, we tested whether the response-threshold of CD4 and CD8 T-cell lines from Vac1 differed from Vac2 and Vac3. To this end, NS3-specific expanded T-cells, isolated 4 weeks prior to HCV infection, were expanded from PBMCs, restimulated with different concentrations of NS3_1258–1272_ ranging from 0.05 to 10 µg/ml, and stained for intracellular IL-2 and IFNγ. Expanded CD8 T-cells from Vac1 were found to respond at a peptide concentration as low as 0.05 µg/ml whereas, at least, 20 times more peptide was required for CD8 T-cells from Vac2 and Vac3 (Figure 5). For IFNγ production by expanded CD4 T-cells the difference is even more striking. Here, we could hardly detect p59-specific IFNγ producing CD4 T-cells in Vac2 and Vac3 while these cells are abundantly present in Vac1.

## Discussion

The chimpanzee is the closest living relative of man and the only validated animal model for HCV infection. The evolutionary proximity of both species is reflected by close genetic and immunological similarity. For these reasons the chimpanzee provides an exquisite animal model, in which immune responses against HCV vaccines and the effect on deliberate infection can be investigated in an experimental setting [2]. Here, we report of an in depth profiling of CD4 and CD8 T-cell responses in four chimpanzees, which were immunized with an experimental vaccine for HCV and subsequently challenged with HCV 1b J4 [11].

While all four animals became infected, only the Patr-A*03∶01 positive animal was able to clear HCV infection. Detailed mapping of CD4 and CD8 T-cell cytokine responses, and the analysis of their cytolytic potential, revealed that expanded T-cells of the vaccinee which effectively cleared HCV infection displayed: a) a higher frequency of peptide-specific IL-2/IFNγ dual cytokine producing CD8 T-cells; b) peptide-specific TNFα production in CD8 T-cells; c) cytolytic capacity against peptide-loaded target cells; d) cytokine induction at a low antigen concentration for the cytolysis-inducing peptide. Remarkably, the cytolysis-inducing peptide p59 was found to bind strongly to Patr-A*03∶01, which was only present in Vac1. Using the NetMHCpan algorithm, it was predicted that peptide LGFGAYMSK, one of the nonamer peptides present in the 15-mer p59, would show the strongest binding to Patr-A*03∶01. Peptide LGFGAYMSK was also predicted to bind to the MHC class I molecules Patr-A*01∶01, A*04∶01, A*04∶04, and A*09∶01, present in all of the studied animals, but only with intermediate affinity between 0.9 and 5 µM. These data are in agreement with the differences in cytokine responses and cytolyses reported in expanded T-cells here and could indicate a particular affinity threshold is necessary for a peptide to induce adequate immune responses for combating HCV. The strong binding of the 15-mer p59 to Patr-A*03∶01 is somewhat unexpected, as generally nonamer peptides bind to MHC class I molecules. Nonetheless, in humans it has been shown that particular MHC class I molecules are capable of binding longer peptides with bulged structures [22], [23].

Stimulation assays would give information about the biological relevance of the binding experiments. However, the limited number of cells stored from these animals, preclude performing such assays.

Unlike *ex vivo* detection of CTL in humans, detection of these cells in chimpanzees can often only be achieved after amplification of antigen specific responses [6], [16]. Obviously, *in vitro* expansion of T-cells from frozen PBMC has its limitations. Antigen presenting cells may be affected by the freezing/thawing process resulting in –partly hampered- antigen presentation. Furthermore, the addition of cytokines and peptides may skew the immune system differently as compared to the *in vivo* situation.

To determine the degranulating capacity of T-cells, CD107a stainings were performed on T-cell lines. However, the results of these assays were unclear and no conclusion could be drawn from it, basically because of the lack of a proper negative control.

Unlike in the intracellular cytokine staining assays, where the difference between cells restimulated with peptide or medium alone was very clear, no clear difference could be observed when CD107a was used. Probably the expansion-protocol used here does agree with antigen specific CD107a staining. Unfortunately, the number of stored cells from these animals is limited and assays like this require too many cells to repeat the assays.

And although we cannot exclude that upon further amplification of antigen specific responses, CTL responses might also be detectable in the other animals, our data suggest that they are of lower magnitude.

To study the possibility of viral escape from immune pressure as a cause of development of chronicity in Vac2, Vac3 and Vac4, analysis of viral sequences was performed and compared with the inoculum sequence (data not shown). Only a limited number of amino acid substitutions were detected in the areas that overlap with cytokine-inducing peptides. There was however no evidence for mutational escape in the immunodominant p59 in any of the animals.

This study highlights a potential role for CD8 mediated cytolytic responses as a discriminatory factor between viral clearance and the development of chronic infection in HCV vaccinated subjects and underscores that this parameter is to be considered as an important immunological readout in future vaccine trials. Indeed, studying peptide binding and CD8 cytolytic responses could complement the other contributing immune mechanisms previously described [11], [24], [25].

It should be noted that the reported data were obtained from peripheral blood and not from the site of HCV-induced inflammation, the liver. For ethical reasons and animal welfare, the number of liver biopsies taken during the study, was limited and not sufficient for the comprehensive analysis as presented in this manuscript. Nevertheless, a direct comparison of CD8 T-cell function between liver and peripheral blood in chimpanzees during HCV infection, showed in essence that the cells in the circulation reflect the situation in the liver [26], [27].

The data presented here, are in line with the finding that self-limiting HCV infection in humans is associated with the presence of HLA-B*57 and HLA-B*27 restricted CD8 T-cell responses [28], [29]. Chimpanzee Patr-A*03∶01 and Patr-B*01∶01 have almost identical B-pocket residues as compared to HLA-B*57. Moreover, peptide binding studies have shown that both Patr-molecules may potentially bind HLA-B*57 or HLA-B*27 [15].

In conclusion, we observed that in contrast to peptide-specific cytokine production, the induction of an effective CD8 T-cell effector function elicited in the context of peptide presentation by appropriate class-I molecules appeared to be associated with clearance of infection.

## Supporting Information

Figure S1 **Gating strategy for the evaluation of cytokine production by CD4 and CD8 cells.** Cells from Vac1, 2 weeks after the last vaccine boost were stimulated with NS3_vaccine_ and restimulated with either NS3_1258–1272_ (p59) or medium alone. Cells within the lymphogate were selected, followed by selection of the CD3 positive, CD20/CD14 negative population, subsequently CD4 and CD8 positive cells were selected. Expression of IFNγ as a function of IL-2 or TNFα was plotted.
(PDF)

Table S1 **Peptide numbering of Vaccine- and Challenge-sequence relative to HCV reference strain H77.**
(PDF)
