# Supplementary figures and images for: Strong Vaccine-Induced CD8 T-Cell Responses Have Cytolytic Function in a Chimpanzee Clearing HCV Infection
Source: PLoS One. 2014 Apr 16;9(4):e95103. doi: 10.1371/journal.pone.0095103 (PMC3989318; doi:10.1371/journal.pone.0095103)

Figure S1

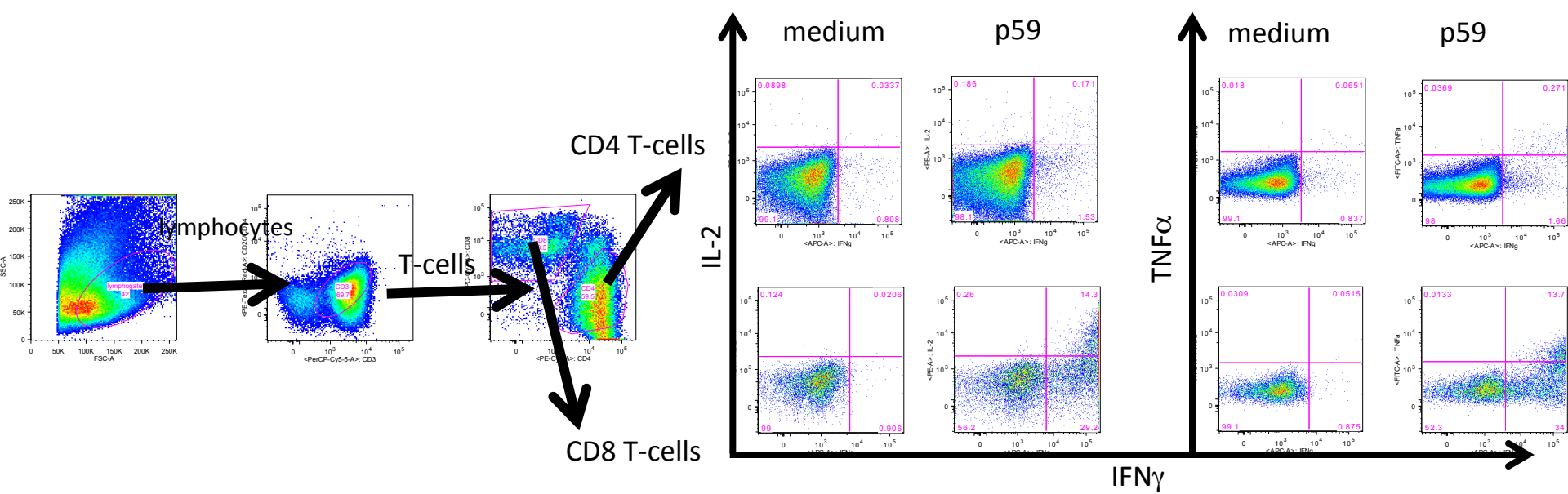

Supplement: Figure S1 — Gating strategy for the evaluation of cytokine production by CD4 and CD8 cells. Cells from Vac1, 2 weeks after the last vaccine boost were stimulated with NS3vaccine and restimulated with either NS31258–1272 (p59) or medium alone. Cells within the lymphogate were selected, followed by selection of the CD3 positive, CD20/CD14 negative population, subsequently CD4 and CD8 positive cells were selected. Expression of IFNγ as a function of IL-2 or TNFα was plotted. (PDF) [file pone.0095103.s001.pdf]
