# Supplementary material for: Strong Vaccine-Induced CD8 T-Cell Responses Have Cytolytic Function in a Chimpanzee Clearing HCV Infection
Source: PLoS One. 2014 Apr 16;9(4):e95103. doi: 10.1371/journal.pone.0095103 (PMC3989318; doi:10.1371/journal.pone.0095103)
Supplement: Table S1 — Peptide numbering of Vaccine- and Challenge-sequence relative to HCV reference strain H77. (PDF) [file pone.0095103.s002.pdf]

Table S1
Peptide numbering of Vaccine- and Challenge-sequence relative to HCV reference strain H77

H77	p#	NS3$_{vaccine}$-sequence	NS3$_{challenge}$-sequence	H77	p#	NS3$_{vaccine}$-sequence	NS3$_{challenge}$-sequence
1026	1	MAPITAYSQQTRGLL	MAPITAYSQQTRGLL	1338	79	TAGARLVVLATATPP	TAGARLVVLATATPP
1030	2	TAYSQQTRGLLGCII	TAYSQQTRGLLGCII	1342	80	RLVVLATATPPGSIT	RLVVLATATPPGSIT
1034	3	QQTRGLLGCIITSLT	QQTRGLLGCIITSLT	1346	81	LATATPPGSITVPHP	LATATPPGSITVPHP
1038	4	GLLGCIITSLTGRDK	GLLGCIITSLTGRDK	1350	82	TPPGSITVPHPNIEE	TPPGSITVPHPNIEE
1042	5	CIITSLTGRDKNQVD	CIITSLTGRDKNQVD	1354	83	SITVPHPNIEEVALS	SITVPHPNIEE**I**ALS
1046	6	SLTGRDKNQVDGEVQ	SLTGRDKNQVDGEVQ	1358	84	PHPNIEEVALSNTGE	PHPNIEE**I**ALSNTGE
1050	7	RDKNQVDGEVQVLST	RDKNQVDGEVQVLST	1362	85	IEEVALSNTGEIPFY	IEE**I**ALSNTGEIPFY
1054	8	QVDGEVQVLSTATQS	QVDGEVQVLSTAT**H**S	1366	86	ALSNTGEIPFYGKAI	ALSNTGEIPFYGKAI
1058	9	EVQVLSTATQSFLAT	EVQVLSTAT**H**SFLAT	1370	87	TGEIPFYGKAIPIEA	TGEIPFYGKAIPIEA
1062	10	LSTATQSFLATCVNG	LSTAT**H**SFLATCVNG	1374	88	PFYGKAIPIEAIKGG	PFYGKAIPIEAIKGG
1066	11	TQSFLATCVNGVCWT	T**H**SFLATCVNGVCWT	1378	89	KAIPIEAIKGGRHLI	KAIPIEAIKGGRHLI
1070	12	LATCVNGVCWTVYHG	LATCVNGVCWTVYHG	1382	90	IEAIKGGRHLIFCHS	IEAIKGGRHLIFCHS
1074	13	VNGVCWTVYHGAGSK	VNGVCWTVYHGAGSK	1386	91	KGGRHLIFCHSKKKC	KGGRHLIFCHSKKKC
1078	14	CWTVYHGAGSKTLAG	CWTVYHGAGSKTLAG	1390	92	HLIFCHSKKKCDELA	HLIFCHSKKKCDELA
1082	15	YHGAGSKTLAGPKGP	YHGAGSKTLAGPKGP	1394	93	CHSKKKCDELAAKLT	CHSKKKCDELAAKLT
1086	16	GSKTLAGPKGPITQM	GSKTLAGPKGPITQM	1398	94	KKCDELAAKLTGLGL	KKCDELAAKLTGLGL
1090	17	LAGPKGPITQMYTNV	LAGPKGPITQMYTNV	1402	95	ELAAKLTGLGLNAVA	ELAAKLTGLGLNAVA
1094	18	KGPITQMYTNVDQDL	KGPITQMYTNVD**L**DL	1406	96	KLTGLGLNAVAYYRG	KLTGLGLNAVAYYRG
1098	19	TQMYTNVDQDLVGWP	TQMYTNVD**L**DLVGWP	1410	97	LGLNAVAYYRGLDVS	LGLNAVAYYRGLDVS
1102	20	TNVDQDLVGWPAPPG	TNVD**L**DLVGWPAPPG	1414	98	AVAYYRGLDVSVIPT	AVAYYRGLDVSVIP**P**
1106	21	QDLVGWPAPPGARSM	**L**DLVGWPAPPGARSM	1418	99	YRGLDVSVIPTSGDV	YRGLDVSVIP**P**SGDV
1110	22	GWPAPPGARSMTPCT	GWPAPPGARSMTPCT	1422	100	DVSVIPTSGDVVVVA	DVSVIP**P**SGDVVVVA
1114	23	PPGARSMTPCTCGSS	PPGARSMTPCTCGSS	1426	101	IPTSGDVVVVATDAL	IP**P**SGDVVVVATDAL
1118	24	RSMTPCTCGSSDLYL	RSMTPCTCGSSDLYL	1430	102	GDVVVVATDALMTGF	GDVVVVATDALMTGF
1122	25	PCTCGSSDLYLVTRH	PCTCGSSDLYLVTRH	1434	103	VVATDALMTGFTGDF	VVATDALMTGFTGDF
1126	26	GSSDLYLVTRHADVI	GSSDLYLVTRHADVI	1438	104	DALMTGFTGDFDSVI	DALMTGFTGDFDSVI
1130	27	LYLVTRHADVIPVRR	LYLVTRHADVIPVRR	1442	105	TGFTGDFDSVIDCNT	TGFTGDFDSVIDCNT
1134	28	TRHADVIPVRRRGDS	TRHADVIPVRRRGDS	1446	106	GDFDSVIDCNTCVTQ	GDFDSVIDCNTCVTQ
1138	29	DVIPVRRRGDSRGSL	DVIPVRRRGDSRGSL	1450	107	SVIDCNTCVTQTVDF	SVIDCNTCVTQTVDF
1142	30	VRRRGDSRGSLLSPR	VRRRGDSRGSLLSPR	1454	108	CNTCVTQTVDFSLDP	CNTCVTQTVDFSLDP
1146	31	GDSRGSLLSPRPVSY	GDSRGSLLSPRPVSY	1458	109	VTQTVDFSLDPTFTI	VTQTVDFSLDPTFTI
1150	32	GSLLSPRPVSYLKGS	GSLLSPRPVSYLKGS	1462	110	VDFSLDPTFTIETTT	VDFSLDPTFTIETTT
1154	33	SPRPVSYLKGSSGGP	SPRPVSYLKGSSGGP	1466	111	LDPTFTIETTTVPQD	LDPTFTIETTTVPQD
1158	34	VSYLKGSSGGPLLCP	VSYLKGSSGGPLLCP	1470	112	FTIETTTVPQDAVSR	FTIETTTVPQDAVSR
1162	35	KGSSGGPLLCPSGHV	KGSSGGPLLCPSGHV	1474	113	TTTVPQDAVSRSQRR	TTTVPQDAVSRSQRR
1166	36	GGPLLCPSGHVVGIF	GGPLLCPSGHVVGIF	1478	114	PQDAVSRSQRRGRTG	PQDAVSRSQRRGRTG
1170	37	LCPSGHVVGIFRAAV	LCPSGHVVGIFRAAV	1482	115	VSRSQRRGRTGRGRS	VSRSQRRGRTGRGRS
1174	38	GHVVGIFRAAVCTRG	GHVVGIFRAAVCTRG	1486	116	QRRGRTGRGRSGIYR	QRRGRTGRGRSGIYR
1178	39	GIFRAAVCTRGVAKA	GIFRAAVCTRGVAKA	1490	117	RTGRGRSGIYRFVTP	RTGRGRSGIYRFVTP
1182	40	AAVCTRGVAKAVDFI	AAVCTRGVAKAVDFI	1494	118	GRSGIYRFVTPGERP	GRSGIYRFVTPGERP
1186	41	TRGVAKAVDFIPVES	TRGVAKAVDFIPVES	1498	119	IYRFVTPGERPSGMF	IYRFVTPGERPSGMF
1190	42	AKAVDFIPVESMETT	AKAVDFIPVESMETT	1502	120	VTPGERPSGMFDSSV	VTPGERPSGMFDSSV
1194	43	DFIPVESMETTMRSP	DFIPVESMETTMRSP	1506	121	ERPSGMFDSSVLCEC	ERPSGMFDSSVLCEC
1198	44	VESMETTMRSPVFTD	VESMETTMRSPVFTD	1510	122	GMFDSSVLCECYDAG	GMFDSSVLCECYDAG
1202	45	ETTMRSPVFTDNSSP	ETTMRSPVFTDNSSP	1514	123	SSVLCECYDAGCAWY	SSVLCECYDAGCAWY
1206	46	RSPVFTDNSSPPAVP	RSPVFTDNSSPPAVP	1518	124	CECYDAGCAWYELTP	CECYDAGCAWYELTP
1210	47	FTDNSSPPAVPQTFQ	FTDNSSPPAVPQTFQ	1522	125	DAGCAWYELTPAETT	DAGCAWYELTPAETT
1214	48	SSPPAVPQTFQVAHL	SSPPAVPQTFQVAHL	1526	126	AWYELTPAETTVRLR	AWYELTPAETTVRLR
1218	49	AVPQTFQVAHLHAPT	AVPQTFQVAHLHAPT	1530	127	LTPAETTVRLRAYLN	LTPAETTVRLRAYLN
1222	50	TFQVAHLHAPTGSGK	TFQVAHLHAPTGSGK	1534	128	ETTVRLRAYLNTPGL	ETTVRLRAYLNTPGL
1226	51	AHLHAPTGSGKSTKV	AHLHAPTGSGKSTKV	1538	129	RLRAYLNTPGLPVCQ	RLRAYLNTPGLPVCQ
1230	52	APTGSGKSTKVPAAY	APTGSGKSTKVPAAY	1542	130	YLNTPGLPVCQDHLE	YLNTPGLPVCQDHLE
1234	53	SGKSTKVPAAYAAQG	SGKSTKVPAAYAAQG	1546	131	PGLPVCQDHLEFWES	PGLPVCQDHLEFWES
1238	54	TKVPAAYAAQGYKVL	TKVPAAYAAQGYKVL	1550	132	VCQDHLEFWESVFTG	VCQDHLEFWESVFTG
1242	55	AAYAAQGYKVLVLNP	AAYAAQGYKVLVLNP	1554	133	HLEFWESVFTGLTHI	HLEFWESVFTGLTHI
1246	56	AQGYKVLVLNPSVAA	AQGYKVLVLNPSVAA	1558	134	WESVFTGLTHIDAHF	WESVFTGLTHIDAHF
1250	57	KVLVLNPSVAATLGF	KVLVLNPSVAATLGF	1562	135	FTGLTHIDAHFLSQT	FTGLTHIDAHFLSQT
1254	58	LNPSVAATLGFGAYM	LNPSVAATLGFGAYM	1566	136	THIDAHFLSQTKQAG	THIDAHFLSQTKQAG
1258	59	VAATLGFGAYMSKAH	VAATLGFGAYMSKAH	1570	137	AHFLSQTKQAGDNFP	AHFLSQTKQAGDNFP
1262	60	LGFGAYMSKAHGIEP	LGFGAYMSKAHGIEP	1574	138	SQTKQAGDNFPYLVA	SQTKQAGDNFPYLVA
1266	61	AYMSKAHGIEPNIRT	AYMSKAHGIEPNIRT	1578	139	QAGDNFPYLVAYQAT	QAGDNFPYLVAYQAT
1270	62	KAHGIEPNIRTGVRT	KAHGIEPNIRTGVRT	1582	140	NFPYLVAYQATVCAR	NFPYLVAYQATVCAR
1274	63	IEPNIRTGVRTITTG	IEPNIRTGVRTITTG	1586	141	LVAYQATVCARAQAP	LVAYQATVCARAQAP
1278	64	IRTGVRTITTGGPIT	IRTGVRTITTGG**S**IT	1590	142	QATVCARAQAPPPSW	QATVCARAQAPPPSW
1282	65	VRTITTGGPITYSTY	VRTITTGG**S**ITYSTY	1594	143	CARAQAPPPSWDQMW	CARAQAPPPSWDQMW
1286	66	TTGGPITYSTYGKFL	TTGG**S**ITYSTYGKFL	1598	144	QAPPPSWDQMWKCLI	QAPPPSWDQMWKCLI
1290	67	PITYSTYGKFLADGG	**S**ITYSTYGKFLADGG	1602	145	PSWDQMWKCLIRLKP	PSWDQMWKCLIRLKP
1294	68	STYGKFLADGGCSGG	STYGKFLADGGCSGG	1606	146	QMWKCLIRLKPTLHG	QMWKCLIRLKPTLHG
1298	69	KFLADGGCSGGAYDI	KFLADGGCSGGAYDI	1610	147	CLIRLKPTLHGPTPL	CLIRLKPTLHGPTPL
1302	70	DGGCSGGAYDIIICD	DGGCSGGAYDIIICD	1614	148	LKPTLHGPTPLLYRL	LKPTLHGPTPLLYRL
1306	71	SGGAYDIIICDECHS	SGGAYDIIICDECHS	1618	149	LHGPTPLLYRLGAVQ	LHGPTPLLYRLGAVQ
1310	72	YDIIICDECHSTDWT	YDIIICDECHSTD**S**T	1622	150	TPLLYRLGAVQNEIT	TPLLYRLGAVQNEI**I**
1314	73	ICDECHSTDWTTILG	ICDECHSTD**S**TTILG	1626	151	YRLGAVQNEITLTHP	YRLGAVQNEI**I**LTHP
1318	74	CHSTDWTTILGIGTV	CHSTD**S**TTILGIGTV	1630	152	AVQNEITLTHPITKF	AVQNEI**I**LTHPITKF
1322	75	DWTTILGIGTVLDQA	D**S**TTILGIGTVLDQA	1634	153	EITLTHPITKFVMAC	EI**I**LTHPITKFVMAC
1326	76	ILGIGTVLDQAETAG	ILGIGTVLDQAETAG	1638	154	THPITKFVMACMSAD	THPITKFVMACMSAD
1330	77	GTVLDQAETAGARLV	GTVLDQAETAGARLV	1642	155	TKFVMACMSADLEVV	TKFVMACMSADLEVV
1334	78	DQAETAGARLVVLAT	DQAETAGARLVVLAT	1646	156	MACMSADLEVVT	MACMSADLEVVT

In red are indicated the amino acids in the NS3challenge sequence that are different from the NS3vaccine sequence
